# Neuroplasticity and Predictors of Alcohol Recovery

**DOI:** 10.35946/arcr.v37.1.10

**Published:** 2015

**Authors:** Dongju Seo, Rajita Sinha

**Affiliations:** Dongju Seo, Ph.D., is an associate research scientist in the Department of Psychiatry; Rajita Sinha, Ph.D., is a Foundations Fund Professor in the Departments of Psychiatry, Neurobiology, and Child Study at Yale University School of Medicine, New Haven, Connecticut.

**Keywords:** Alcohol use, abuse, and dependence, alcoholism, alcohol effects and consequences, alcohol-related neuroadaptations, neurobiology, brain, neuroplasticity, prefrontal-striatal-limbic circuit, autonomic nervous system, hypothalamic-pituitary-adrenal axis system, recovery, relapse

## Abstract

Chronic alcohol-related neuroadaptations in key neural circuits of emotional and cognitive control play a critical role in the development of, and recovery from, alcoholism. Converging evidence in the neurobiological literature indicates that neuroplastic changes in the prefrontal–striatal–limbic circuit, which governs emotion regulation and decisionmaking and controls physiological responses in the autonomic nervous system and hypothalamic–pituitary–adrenal axis system, contribute to chronic alcoholism and also are significant predictors of relapse and recovery. This paper reviews recent evidence on the neuroplasticity associated with alcoholism in humans, including acute and chronic effects, and how these neurobiological adaptations contribute to alcohol recovery, along with the discussion of relevant clinical implications and future research directions.

Recovery from alcoholism is a complex and long-term process with high relapse rates. Therefore, understanding why people relapse has been critically important to improving treatment outcomes. To that end, researchers are looking for clinical and biological markers that predict relapse after treatment and to use those risk factors to develop effective treatments to reduce relapse rates. One promising research area is examining how alcohol changes structure and function in the brain, affecting what neuroscience calls neuroplasticity and causing neuroadaptations that can affect the brain’s reward and decision-making centers and, in turn, affect relapse and recovery.

During recovery, individuals with alcohol use disorder (AUD) psychologically and physiologically recuperate from the deleterious effects of alcohol exposure by achieving complete abstinence or low-level, nonhazardous alcohol intake. The National Epidemiologic Survey of Alcohol-Related Conditions (NESARC) conducted 43,093 in-person interviews with a national sample of adults and found that 4,422 subjects, at some point prior to the past year, met the criteria for alcohol dependence, as defined by the *Diagnostic and Statistical Manual of Mental Disorders, Fourth Edition* (DSM–IV). According to the survey, during the preceding year, of those 4,422 alcohol-dependent people, 35.9 percent achieved either low-risk drinking (17.7 percent) or abstinence (18.2 percent) (Dawson et al. 2005). The study also noted that recovery rates tend to be even lower in clinical samples of people with severe dependence and for people with lifetime dependence or at high risk of relapse (Dawson et al. 2005). Additionally, the risk of relapse after treatment for AUD increases if people have concurrent conditions, such as anxiety or stress sensitivity (Kushner et al. 2005; Sinha et al. 2011).

In an effort to identify clinical and biological markers that predict relapse risk, researchers have looked toward the brain and alcohol-related changes in the brain that might make it more difficult for people with AUD to recover successfully. In particular, recent research has capitalized on advances in neuroimaging techniques to examine neuroplastic changes that may increase vulnerability to alcoholism and alcohol relapse (Buhler and Mann 2011). In fact, evidence suggests that chronic, heavy alcohol consumption is related to neuronal changes that target critical central nervous system (CNS) functions governing homeostasis, emotion regulation, and decisionmaking. These changes, in turn, may make it significantly more challenging for people to stop drinking and may result in various comorbid, psychological, and physiological symptoms (Bechara 2005; Breese et al. 2011). For instance, when people with AUD are abstinent, altered neural circuits of stress and reward modulation make them highly sensitive to stress and increase alcohol craving and other withdrawal symptoms, including anxiety, negative emotion, autonomic nervous system (ANS) disruption, fatigue, and sleep problems (Breese et al. 2011; Seo and Sinha 2014).

These chronic alcohol-related neuronal changes and their co-occurring symptoms, such as stress, may serve as markers of alcohol relapse and long-term recovery but are not currently addressed in most AUD treatment programs. Already there is evidence that people who maintain long-term abstinence show functional differences in resting-state brain synchrony relative to those with short-term abstinence (Camchong et al. 2013).

This paper reviews the evidence for neuronal changes associated with alcoholism in humans, including those resulting from acute and chronic effects of alcohol, and how these changes contribute to alcohol relapse. To help understand alcohol recovery in a clinical research setting, the review will specifically focus on neuroplastic changes associated with alcohol relapse immediately following treatment. This paper also reviews the effects of stress on alcohol-related neuroplasticity and alcohol recovery, along with relevant clinical implications and future research directions. Elucidating the link between neuroplastic changes and alcohol recovery will contribute to our understanding of complex alcohol-related symptomatology and provide insights into the development of effective treatments to improve recovery from alcoholism.

## Neuroplastic Changes in the PSL Circuit

Neuroplasticity refers to changes in the nervous system that occur in response to various stimuli or experiences and include structural and functional re-organization (Sale et al. 2014). These neuroplastic changes can be acute or take place over time (Sale et al. 2014) and can either be positive or negative, depending on the experience (Vance and Wright 2009). Neuroplastic changes in response to alcohol or other addictive substances are most commonly regarded as negative neuroplasticity associated with suboptimal functioning and maladaptive behaviors (Kalivas and O’Brien 2008). Addiction researchers frequently use the term “neuroadaptation” when referring to alcohol- or drug-related neuroplastic changes in the CNS (Breese et al. 2011; Cohen 2003; Shaham and Hope 2005). Thus, the addiction neuroscience literature uses the concepts of neuroadaptation and neuroplasticity interchangeably.

In studies of alcoholism, substantial evidence indicates short-term and long-term pharmacological effects of alcohol on the nervous system and related neurophysiological dysfunction (Seo and Sinha 2014). Specifically, research has well documented acute and chronic alcohol-related neuroadaptations in the prefrontal–striatal–limbic (PSL) circuit, which helps modulate motivation and emotion (Buhler and Mann 2011). The circuit consists of the striatal-limbic system, which is involved in the brain’s reward system in the striatum, and its stress system, in the amygdala; and the prefrontal regulatory region, which includes the medial prefrontal cortex (PFC), the anterior cingulate cortex (ACC), the orbitofrontal cortex (OFC), and the dorsolateral PFC. As a whole, the PSL circuit plays a pivotal role in modulating reward, stress, and decision-making throughout the course of alcoholism, including the disorder’s initial development, alcohol dependence, uncontrollable alcohol seeking, and continued alcohol relapse despite its negative consequences (Seo and Sinha 2014).

One way this circuit could interact with alcohol to influence these phases of alcoholism is through a part of the PSL circuit called the ventromedial prefrontal cortex (VmPFC), a brain region critical for emotional and behavioral control and that regulates the ANS and hypothalamic–pituitary–adrenal (HPA) axis systems (Radley 2006). If repeated alcohol use disturbs the VmPFC, it could disrupt the regulation and homeostasis of ANS and HPA axis system functioning, which can result in high physiological and emotional arousal, and, in turn, is associated with high alcohol craving (Sinha et al. 2009). Continued chronic alcohol-related changes in the PSL circuit could place individuals in a neurobiologically vulnerable state, substantially compromising their ability to control the urge to drink heavily and increasing the risk that they will resume drinking after a period of abstinence. For this reason, researchers have suggested that maintaining an intact PSL circuit is critical to a person’s ability to overcome alcohol seeking and relapse urge (Koob 2009; Seo and Sinha 2014; Sinha 2008). Thus, understanding acute and chronic neuroadaptive patterns in the course of alcohol illness, especially in the PSL circuit, may provide insights into alcohol-related clinical symptoms, emotional and behavioral changes, and the potential impact of these patterns on alcohol recovery.

## Acute Effects of Alcohol on Brain Response

Alcohol has clear and immediate pharmacological effects on the brain (see for example, Wallner and Olsen 2008). Specifically, neuroimaging studies of acute alcohol consumption in healthy social drinkers find specific effects on emotional processing and modulation (Gilman et al. 2008), cognitive disruption (Soderlund et al. 2007), and decisionmaking (Gilman et al. 2012).

In relation to emotional processing and modulation, several functional magnetic resonance imaging (fMRI) studies report acute effects of alcohol on reducing anxious and negative emotion and increasing alcohol craving by modulating limbic-striatal activity. In one study (Gilman et al. 2008), researchers administered an intoxicating dose of alcohol to healthy individuals via intravenous injection and found they had reduced limbic response to fearful faces and increased striatal activity. In another study (Sripada et al. 2011), researchers found decreased activity in the amygdala when 12 healthy but heavy social drinkers ingested alcohol and then viewed socioemotional stimuli, including fearful/angry faces. Another study (Gorka et al. 2013), using the same study sample and design, found that drinking alcohol reduced the connectivity between the amygdala and orbitofrontal cortex, suggesting that the regulatory part of the brain is interacting less with the amygdala during the processing of socioemotional stimuli under the influence of alcohol. When a different group of healthy but heavy social drinkers received an alcohol taste cue, researchers saw increased activity in the VmPFC, the ACC, and the ventral striatum (Filbey et al. 2008). Consistent with this, researchers saw enhanced activity in regions of the ventral and dorsal striatum in healthy male social drinkers asked to imagine an alcohol cue–related situation, with a significant correlation between alcohol craving and activity in these regions (Seo et al. 2011).

Several fMRI studies also have reported an influence of alcohol on cognitive function and decisionmaking. Alcohol consumption in healthy individuals resulted in impaired episodic memory encoding, which, in turn, was associated with reduced activity in the lateral PFC (Soderlund et al. 2007). In addition, during a decision-making task, acute alcohol administration via intravenous injection increased risk-taking behaviors, increased striatal reactivity to risk choices, and blunted brain response to emotional feedback related to both winning and losing (Gilman et al. 2012).

Taken together, neuroimaging studies demonstrate the significant influence of alcohol in healthy individuals via alterations in the PSL circuit, including reduced limbic response to negative emotional stimuli (Gilman et al. 2008; Sripada et al. 2011), enhanced striatal response to rewarding stimuli (Filbey et al. 2008; Gilman et al. 2008; Seo et al. 2011) and to risky decision-making (Gilman et al. 2012), and impaired episodic memory functioning (Soderlund et al. 2007). These studies clearly point to the PSL circuit as a critical early target of alcohol effects and its potential, deleterious impact on neuroplasticity with chronic alcohol abuse.

## Neuroadaptations, Chronic Alcoholism, and Recovery

Not surprisingly, just as acute alcohol consumption affects the brain, so does chronic, heavy alcohol consumption. In fact, studies consistently report alcohol-related neuroadaptive changes in the PSL circuit, along with related allostatic changes in physiological functions, including ANS and HPA axis systems (Breese et al. 2011; Seo and Sinha 2014). The brain regions affected include the reward system, the stress system, and the prefrontal regulatory system (Seo and Sinha 2014).

### Reward System Dysfunction and Alcohol Recovery

Several lines of research link changes in the striatum and, therefore, the brain’s reward system to chronic alcohol use:

- Blunted dopamine release and other types of dopamine dysfunction in the striatum may be a biomarker indicating increased vulnerability to alcohol and other substance use (Trifilieff and Martinez 2014);
- Chronic alcohol abuse and exposure result in alterations in reward brain regions, such as the ventral striatum, leading to disrupted dopamine transmission and striatal activity (Martinez et al. 2005; Seo and Sinha 2014; Volkow et al. 2002).
- Detoxified AUD patients show signs of altered reward responses, such that enhanced ventral striatal activity is more biased toward alcohol cues than other reward cues, such as money (Wrase et al. 2007); and
- People with AUD had reduced levels of dopamine D2 receptors in their frontal-striatal regions compared with control subjects (Volkow and Fowler 2000).

Researchers have suggested that repeated alcohol use gradually enhances incentive salience and craving response toward alcoholic beverages by altering the reward pathways and triggering more alcohol craving and drug seeking (Breese et al. 2011; Robinson and Berridge 1993). Altered reward-system function, in turn, could further aggravate a lack of control over the reward response and intensify alcohol craving and the urge to drink alcohol, both of which are associated with increased vulnerability to alcohol relapse (Breese et al. 2011; Sinha 2008). Several lines of research support this theory, reporting significant associations between altered striatal response and alcohol relapse:

- Decreased levels of striatal dopamine D2 receptor persisted in AUD patients and did not recover up to 4 months after alcohol detoxification (Volkow et al. 2002).
- Patients who relapsed within 3 months after discharge had lower levels of dopamine during detoxification than patients who did not relapse, according to a study that measured dopamine in 21 AUD inpatients using [^123^I] iodobenzamide (IBZM) single-photon emission computerized tomography (SPECT) (Guardia et al. 2000).
- AUD patients who relapsed within 3 months of becoming abstinent showed increased activity in part of the striatum, called the ventral putamen, when viewing visual alcohol cues during the early weeks of abstinence (at least 1 week after detoxification) (Braus et al. 2001).
- On the other hand, recently detoxified (1 to 3 weeks) alcoholic patients with a blunted striatal response to positive emotional pictures relative to neutral pictures had a greater number of drinking days and a higher amount of alcohol consumed during the 6-month followup (Heinz et al. 2007).

These studies suggest that striatal reward system function plays a key role in the development of alcoholism and continues to influence the course of alcoholism by affecting alcohol recovery. Continued alcohol use seems to sensitize striatal reward function and increase incentive salience toward alcohol stimuli. In AUD patients, this altered striatal system may further intensify craving responses and trigger withdrawal symptoms during alcohol-free periods, increasing risk for relapse (Vanderschuren and Pierce 2010).

### Neuroadaptations in the Neural Circuit of Stress Modulation

As excessive alcohol use continues, alterations in the reward system could result in allostatic changes in other brain regions closely connected with the striatum, including the limbic regions and the PFC (Breese et al. 2011; Koob and Volkow 2010). In particular, alterations in the stress system may play a crucial role in the well-known comorbid symptoms associated with alcohol dependence, including aversive emotional states such as anxiety, negative mood, high stress sensitivity, and stress-induced alcohol craving (for example, see Sinha et al. 2009).

Stress is a critical factor in increasing alcohol craving and compulsive alcohol consumption (Breese et al. 2005; Koob 2009), as evidenced by both preclinical and clinical studies, including overconsumption of alcohol in male mice with prenatal stress (Campbell et al. 2009), early trauma associated with greater alcohol use and alcohol craving (Schumache et al. 2006), and increased alcohol use after the 9/11 terrorist attacks among New York City residents (Vlahov et al. 2006). Individuals suffering from chronic alcoholism frequently report high stress sensitivity and stress-triggered intense craving (Fox et al. 2007; Sinha et al. 2009). And stress sensitivity plays a crucial role in increased alcohol craving to alleviate aversive emotions or stimuli (Gilpin and Koob 2008)—known as “negatively reinforced craving”—which becomes a main driving force for drinking as excessive alcohol use continues (Koob 2009; Koob et al. 2004; Sinha 2008).

The brain’s stress response involves activation of the ANS and HPA axis systems to promote regulation of physiological arousal and also facilitate adaptive coping (Sinha 2008). Chronic alcoholism is associated with impaired autonomic regulation characterized by high basal heart rate, reduced heart rate variability, and increased blood pressure (Quintana et al. 2013; Sinha et al. 2009; Stormark et al. 1998; Thayer et al. 2006). Further, upregulated HPA axis function, including elevated levels of basal cortisol and adreno-corticotrophic hormone (ACTH), has been frequently found in people with AUD (Breese et al. 2011; Sinha 2008; Sinha et al. 2009). Consistent with this, alcoholics who continue to drink, and those experiencing withdrawal symptoms, have increased levels of basal stress hormones, including cortisol, norepinephrine, and corticotropin-releasing factor (CRF) (for review, see Breese et al. 2011). In addition, a study of 93 treatment-engaged, 1-month-abstinent AUD patients found strong associations between alcohol relapse and HPA axis system function. In this study, greater morning adrenal sensitivity indexed by the cortisol-to-ACTH ratio significantly predicted a shorter time to future initial relapse as well as heavy-drinking relapse (Sinha et al. 2011), indicating a significant role of chronic alcohol-related stress pathology in alcohol recovery.

In terms of brain regions involved, researchers postulate that neuroadaptations in the amygdala may influence negatively reinforced craving and alcohol seeking (Koob 2009; Koob et al. 2004; Sinha 2008). The amygdala is involved in stress-induced physiological responses via modulation of CRF and norepinephrine pathways, which are well known for their contribution to negative reinforcement aspects of addiction (Koob 1999, 2009). Research with AUD patients abstinent for 1 week shows a potential role of altered amygdala functioning in alcohol recovery. In this study, patients who relapsed had reduced amygdala volume compared with patients who did not relapse, and the reduction of the amygdala volume was significantly associated with alcohol craving and the amount of follow-up alcohol drinking (Wrase et al. 2008). Consistent with these data, preclinical studies report associations between altered response in the extended amygdala and stress-primed drug reinstatement (for review, see Kalivas and McFarland 2003).

**Figure 1:**
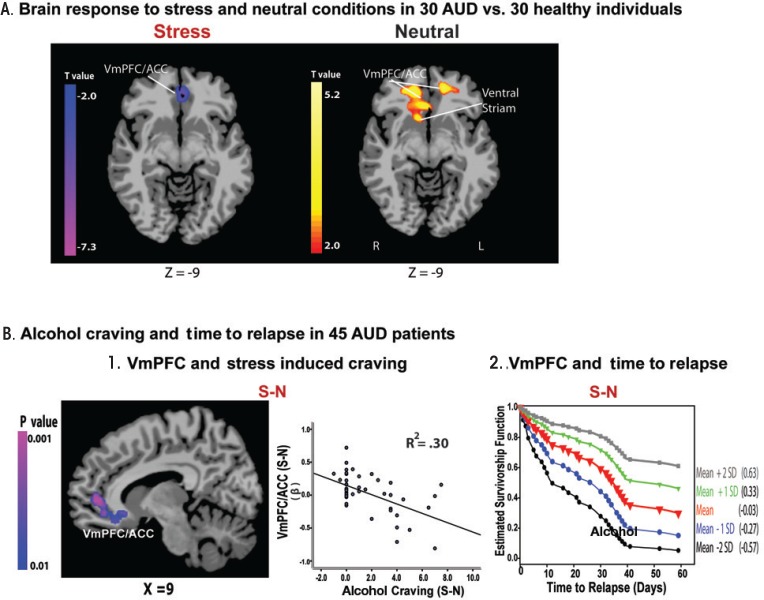
Hypoactive ventromedial prefrontal cortex (VmPFC) response to stress, alcohol craving, and relapse risk. **(A)** Hypoactive VmPFC response to stress but hyperactive response to neutral-relaxing condition in 30 patients with alcohol use disorder (AUD) compared with 30 healthy control subjects. AUD patients showed hypoactive VmPFC and anterior cingulate cortex (ACC) response to stress compared with demographically matched healthy control subjects (*P* < 0.05; whole-brain familywise error correction [FWE] corrected). **(B)** Neural correlates of alcohol craving and relapse in 45 AUD patients. **(B-1)** Whole-brain correlation analyses indicated that hypoactive VmPFC/ACC response to stress, compared with a neutral condition, was associated with increased alcohol craving during stress (*r* = −0.55; *R^2^* = 0.30; *P* < 0.01 whole-brain FWE corrected). No other regions were significantly associated with craving in this whole-brain voxel-based analysis. **(B-2)** Estimated survival functions for time to initial alcohol relapse are presented to illustrate the increasing risk of relapse with signal changes in the VmPFC hypoactivity during stress relative to the neutral condition: mean (in red) +1 (green) and +2 (gray) standard deviation (SD) above the mean, and −1 (blue) and −2 (black) SD below the mean. Cox proportional hazards regression analysis also indicates that hypoactive response during stress-neutral predicted a shorter time to initial alcohol use (χ^2^ = 5.37, *P* < 0.05; hazard ratio [HR] = 0.22, confidence interval [CI] = 0.06–0.79) as well as heavy-drinking relapse (χ^2^ = 5.5, *P* < 0.05; HR = 0.21, CI = 0.06–0.77). S-N = stress-neutral. NOTE: This figure is reproduced with the permission of the American Medical Association (Seo et al. 2013).

During stress, ANS and HPA axis function are under the regulatory control of the VmPFC (Figueiredo et al. 2003; Radley 2006). Preclinical studies demonstrate decreased HPA axis response to stress following VmPFC lesions (Radley 2006) and find that the VmPFC maintains stress-related inhibitory control over HPA axis arousal (Figueiredo et al. 2003). In addition, a meta-analysis of studies in humans reported significant associations between brain activity in the VmPFC and amygdala and ANS function indexed by heart rate variability (Thayer et al. 2012). Given that chronic alcoholism is associated with HPA axis and ANS system dysfunctions, as discussed earlier, these findings on the VmPFC regulation over stress-related HPA axis and ANS arousal suggest that individuals with chronic alcoholism may have underlying VmPFC dysfunction in response to stress. Consistent with this hypothesis, a recent fMRI study (Seo et al. 2013) found lowered activity in the stress modulatory regions involving VmPFC/ACC during stress exposure in 30 AUD patients engaged in inpatient treatment and abstinent for 4 weeks, compared with 30 matched healthy control subjects (figure 1A). Interestingly, the researchers observed an opposite pattern when the subjects were relaxed: AUD patients showed hyperactive VmPFC/ACC compared with control subjects (figure 1A). More importantly, to prospectively assess relapse and early recovery, these researchers followed the same 30 AUD patients, plus 15 others, after they completed inpatient treatment. Results indicated that lowered VmPFC activity in response to stress exposure relative to the response when patients were relaxed was significantly associated with stress-induced alcohol craving and also predicted a shorter time to future relapse (see figure 1B) (Seo et al. 2013). In addition, lower VmPFC activity and insula response to stress was significantly correlated with more days of alcohol use during subsequent followup, emphasizing the contribution of altered stress neural circuitry to relapse susceptibility (Seo et al. 2013).

Although further work still is needed to fully understand the associations between stress-related neural response during abstinence, treatment, and early alcohol recovery, available data suggest that neuroadaptations in the peripheral and CNS involved in stress modulation play a significant role in recovery from chronic alcoholism. Altered emotional and stress responses and poor abilities to cope under stress observed in people with AUD may increase vulnerability to high-stress–related craving, relapse, and alcohol drinking, especially under challenging life circumstances.

### PFC Regulatory Function in Alcohol Recovery

If repeated alcohol exposure disrupts the limbic-striatal system, the result could progressively debilitate prefrontal executive functions (Seo and Sinha 2014). Chronic alcohol-related PFC impairments, in turn, can compromise one’s ability to recover from alcoholism by adversely influencing executive function, inhibitory control, and decisionmaking (Bechara 2005; Goldstein and Volkow 2011). Many neuroimaging studies consistently have indicated structural and functional deficits in prefrontal regulatory regions associated with chronic alcoholism (for review, see Buhler and Mann 2011).

Structural imaging studies, for example, find reduced gray matter volume in the medial PFC/OFC and its surrounding regions in AUD patients, and this is associated with poor treatment outcome. One study (Durazzo et al. 2011) examined cortical thickness in people with AUD who averaged 35 to 36 years of lifetime drinking and were seeking treatment. Patients who relapsed by a 12-month followup had decreased cortical thickness, especially in the OFC and right rostral/caudal middle frontal cortex, compared with AUD patients who continued to abstain after 12 months. (Durazzo et al. 2011). In an MRI study that examined AUD patients with an average of 18.6 years of alcohol use, soon after they became abstinent during treatment, patients had decreased brain volume in the gray matter of their medial PFC and posterior parietal-occipital area. At a 3-month followup after treatment, the researchers found that the degree of volume reductions significantly predicted a shorter time to initial relapse as well as heavy-drinking relapse, even after controlling for years of alcohol use and baseline alcohol intake (Rando et al. 2011). Furthermore, a study investigating both structural and functional patterns in detoxified AUD patients found that patients who relapsed within 3 months after completing treatment showed atrophy in regions of OFC and right medial PFC and ACC compared with patients who did not relapse. Relapsed patients also showed increased alcohol cue–induced activation in the left medial PFC regions in this study (Beck et al. 2012).

Functional neuroimaging studies also have reported connections between alcoholism, alcohol recovery, and altered activity in the medial PFC, OFC, and striatum. In AUD individuals, PET imaging studies found decreased glucose metabolism in the frontal cortex during alcohol withdrawal (for review, see Volkow and Fowler 2000) and reduced availability in striatal D2 receptors associated with lowered OFC/ACC function (Volkow et al. 2007). A study performing a 3-month followup on alcohol-dependent patients who had been abstinent for an average of 7 weeks prior to the start of the study, found that the five patients who relapsed showed pronounced activity in the medial PFC, ACC, and striatum when they viewed alcohol pictures. In these patients, there was a significant association between medial PFC activity and the amount of subsequent alcohol consumption (Grusser et al. 2004). Another study used SPECT to study brain blood flow in AUD inpatients at the end of an alcohol detoxification program that lasted at least 7 days. The nine patients who relapsed 2 months later displayed decreased blood flow in the medial frontal lobe and poor working-memory performance relative to 11 abstainers. The working-memory deficits were associated with low blood flow in the medial frontal lobe (Noel et al. 2002). These neuroimaging studies point to a potential significant role of structural and functional neuroplasticity in the prefrontal regulatory regions involving the medial PFC, OFC, and ACC in increased relapse risk and poor alcohol recovery.

### Neuronal Hyperexcitability and Alcohol Recovery

**Figure 2:**
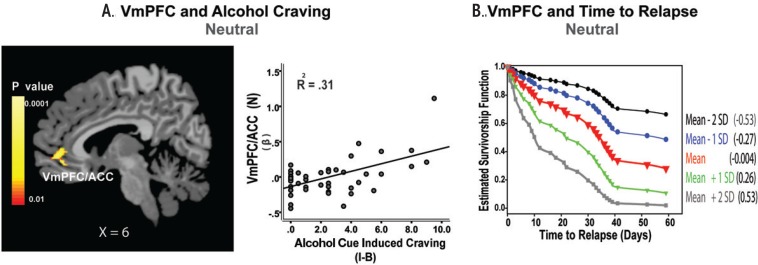
Hyperactive ventromedial prefrontal cortex (VmPFC) response to the neutral-relaxing condition, alcohol craving, and relapse risk. **(A)** In 45 patients with alcohol use disorder (AUD), hyperactive response in the VmPFC and anterior cingulate cortex (ACC) when they are exposed to neutrally relaxing situations during brief guided imagery was significantly associated with high alcohol craving during alcohol cue imagery (*R* = 0.56; *R* ^2^ = 0.31, *P* < 0.01 whole-brain FWE corrected). **(B)** Estimated survival functions for time to initial alcohol relapse, showing that the more VmPFC hyperactivity during the neutral condition, the shorter the time to subsequent initial relapse and heavy drinking relapse: mean (in red) +1 (green) and +2 (gray) standard deviation (SD) above the mean, and −1 (blue) and −2 (black) SD below the mean. Cox proportional hazards regression analysis indicates that hyperactive VmPFC response during the neutral condition predicted a shorter time to initial alcohol use (χ^2^ = 6.39, *P* = 0.01; hazard ratio [HR] = 8.45, confidence interval [CI] = 1.6–44.2) as well as heavy drinking relapse (χ^2^ = 7.39, *P* < 0.01, HR = 8.68, CI = 1.8–41.2). I-B = imagery minus baseline ratings. SOURCE: This figure is reproduced with the permission of the American Medical Association (Seo et al. 2013).

Recent evidence in humans suggests that excessive, chronic alcohol consumption may lead to hyperexcitability of neurons in the CNS, which, in turn, plays a role in alcohol addiction and recovery (Porjesz and Begleiter 2003; Seo et al. 2013; Sinha et al. 2011). Indeed, studies have found hyperactive CNS and electroencephalogram (EEG) responses in people with AUD, including increased excitatory neurotransmission associated with long-term alcohol use and hyperactive EEG responses in the frontal regulatory regions (Bauer 2001; Porjesz and Begleiter 2003). Studies also have found that alcohol-related neuronal adaptations on basal-state physiology, including upregulated ANS and HPA axis systems, underlie high alcohol craving, poor clinical outcome, and relapse vulnerability by disrupting physiological arousal (Breese et al. 2011; Seo et al. 2013; Sinha et al. 2011). In addition, a recent fMRI study found significant associations between hyperactive brain response during a relaxed state and susceptibility to alcohol craving and relapse in AUD patients who were engaged in treatment and 4 to 8 weeks abstinent. In this study, hyperactivity during relaxation in the VmPFC/ACC, but no other region, was associated with greater alcohol craving when subjects were presented with alcohol cues (figure 2A). In addition, the VmPFC/ACC hyperactivity predicted a shorter time to subsequent initial relapse and heavy-drinking relapse (figure 2B), as well as more alcohol use during a 90-day follow-up period (Seo et al. 2013). These findings highlight the important role of basal-state hyperactivity and integrity of VmPFC function in recovery from chronic alcoholism.

## Conclusion and Future Directions

Alcoholism is a chronic illness, characterized by high relapse risk. Research now suggests that underlying this chronic relapse risk may be negative neuroplastic changes in the brain caused by the cycle of continued alcohol abuse and repeated brief alcohol abstinence and/or alcohol withdrawal. These neuroplastic changes occur in the PSL circuit, which regulates emotions and decisionmaking, which, in turn, influence alcohol recovery (Bechara 2005; Everitt and Robbins 2005; Goldstein and Volkow 2011). Within the PSL circuit, the PFC regulates limbic and striatal regions to modulate emotional and physiological responses to various reward- and stress-related stimuli (Seo and Sinha 2014). In individuals with chronic alcoholism, persistent sensitization of subcortical limbic-striatal regions from prolonged alcohol use could compromise the PFC regulatory function, resulting in difficulties in emotional regulation, poor impulse control, and high alcohol craving. Substantially weakened PFC function could, in turn, further disinhibit limbic-striatal responses especially under challenging situations, including stress and exposure to alcohol-related cues. In addition, given the crucial role of the PFC in inhibitory control and decisionmaking (Bechara 2005; Goldstein and Volkow 2011), altered PFC function could result in an inability to inhibit compulsive alcohol seeking and poor decisionmaking when confronted with the choice to return to drinking and continued alcohol use despite negative consequences, thereby aggravating the relapse cycle.

The evidence supporting a role of the neuroadaptative changes in the PSL circuit in alcohol recovery points to important clinical implications:

- The neuroadaptive patterns in this circuit may serve as a set of neurobiological markers of alcohol relapse and recovery. Future research can validate these patterns and investigate their use to help predict relapse vulnerability and to identify people with the greatest challenge to alcohol recovery in the clinical setting.
- Researchers could develop and test novel treatment strategies that target these validated biomarkers and attempt to reverse these neuroadaptations and significantly improve the chances of recovery from alcoholism. Already evidence supports a mediating role of neuroplasticity in the PSL circuit in improving treatment outcome. A study (Muller et al. 2009) with deep brain stimulation showed the effectiveness of this method in recovering ventral striatal function in AUD individuals. In addition, a recent fMRI study (Brewer et al. 2011) showed that meditators with mindfulness training experience have stable VmPFC and posterior cingulate cortex activity compared with control subjects as well as stronger connectivity between cingulate cortex and dorsolateral PFC, suggesting that mindfulness training may hold potential for treating alcoholism. Consistent with these data, a recent clinical outcome study (Bowen et al. 2014) reported that participants assigned to a mindfulness-based relapse prevention program had fewer days of drug use and decreased heavy drinking, compared with cognitive–behavioral relapse prevention or 12-step–based program approaches at a12-month followup.
- Researchers could develop treatments that target withdrawal symptoms and stress-related pathology, such as stress-induced craving and alcohol seeking, implicated by the alcohol-related neuroadaptations in the PSL circuit. For instance, alpha1-adrenergic antagonists, such as Prazosin, show promise for improving stress-induced deficits and impaired PFC function from chronic stress (for a review, see Arnsten 2009). This drug also reduces alcohol withdrawal symptoms and stress-related alcohol seeking in animals (Kukolja et al. 2011; Walker et al. 2008) and improves stress and alcohol cue–induced craving and alcohol use outcomes in humans (Fox et al. 2012; Simpson et al. 2009). Alternative medicines, such as herbal remedies, are another area of interest. For example, Ge Gen (Kudzu root, Rx. Pueriariae), an herbal remedy frequently used in Eastern medicine, is effective in controlling alcohol intake and alcohol-related withdrawal symptoms (Benlhabib et al. 2004; Lukas et al. 2005). Studies that examine whether the treatments can restore PSL circuit function, especially the VmPFC, and improve alcoholism recovery rates, would be beneficial.

In conclusion, current neurobiological research in humans has identified neuroplasticity in the PSL circuit and its related dysfunctions as key factors increasing relapse risk and jeopardizing alcohol recovery. Further development of biomarkers for these alcohol-related neuroadaptive changes and new treatments that aim to restore the brain have the potential to influence the development of new treatment strategies to promote alcohol recovery and reduce the global burden associated with alcoholism.
